# LIMK2-1 Is a Phosphorylation-Dependent Inhibitor of Protein Phosphatase-1 Catalytic Subunit and Myosin Phosphatase Holoenzyme

**DOI:** 10.3390/ijms26157347

**Published:** 2025-07-30

**Authors:** Andrea Kiss, Emese Tóth, Zsófia Bodogán, Mohamad Mahfood, Zoltán Kónya, Ferenc Erdődi

**Affiliations:** Department of Medical Chemistry, Faculty of Medicine, University of Debrecen, H-4032 Debrecen, Hungary; kissand@med.unideb.hu (A.K.); toth.emese@med.unideb.hu (E.T.); bodogan.zsofia@med.unideb.hu (Z.B.); mahfood.mohamad@med.unideb.hu (M.M.); konya.zoltan@med.unideb.hu (Z.K.)

**Keywords:** LIM kinase 2 (LIMK2), LIMK2 isoform 1 (LIMK2-1), protein phosphatase-1 (PP1), C-kinase-activated protein phosphatase-1 (PP1) inhibitor of 17 kDa (CPI-17), myosin phosphatase holoenzyme (MP), calyculin A (CLA)

## Abstract

The C-kinase-activated protein phosphatase-1 (PP1) inhibitor of 17 kDa (CPI-17) is a specific inhibitor of the PP1 catalytic subunit (PP1c) and the myosin phosphatase (MP) holoenzyme. CPI-17 requires the phosphorylation of Thr38 in the peptide segment ^35^ARV(P)TVKYDRREL^46^ for inhibitory activity. CPI-17 regulates myosin phosphorylation in smooth muscle contraction and the tumorigenic transformation of several cell lines via the inhibition of MP. A phosphospecific antibody (anti-CPI-17^pThr38^) against the phosphorylation peptide was used to determine the phosphorylation levels in cells. We found that phospho-CPI-17 and its closely related proteins are not present in HeLa and MCF7 cells after inducing phosphorylation by inhibiting phosphatases with calyculin A. In contrast, cross-reactions of proteins in the 40–220 kDa range with anti-CPI-17^pThr38^ were apparent. Searching the protein database for similarities to the CPI-17 phosphorylation sequence revealed several proteins with 42–75% sequence identities. The LIMK2-1 isoform emerged as a possible PP1 inhibitor. Experiments with Flag-LIMK2-1 overexpressed in tsA201 cells proved that LIMK2-1 interacts with PP1c isoforms and is phosphorylated predominantly by protein kinase C. Phosphorylated LIMK2-1 inhibits PP1c and the MP holoenzyme with similar potencies (IC50 ~28–47 nM). In conclusion, our results suggest that LIMK2-1 is a novel phosphorylation-dependent inhibitor of PP1c and MP and may function as a CPI-17-like phosphatase inhibitor in cells where CPI-17 is present but not phosphorylated upon phosphatase inhibition.

## 1. Introduction

The C-kinase-activated protein phosphatase-1 (PP1) inhibitor of 17 kDa (CPI-17) was first isolated from smooth muscle [1] and identified as a specific inhibitor of the PP1 catalytic subunits (PP1c) and the myosin phosphatase (MP) holoenzyme. MP consists of a PP1cδ catalytic subunit isoform and a 130/133 kDa myosin phosphatase target subunit (MYPT). The MYPT1 isoform of MYPT is present in most cells [2]. Phosphorylation of Thr38 in CPI-17 is required for inhibitory activity. In addition to protein kinase C (PKC), numerous other kinases may phosphorylate this inhibitory site [3,4]. CPI-17 is expressed predominantly in tonic and phasic smooth muscle and brain tissue, but not in adult cardiac or skeletal muscles. CPI-17 is also present in platelets and epithelial and endothelial cells [5].

Detailed biochemical and structural analyses proved that the central region of CPI-17 (residues 35–120), including Thr38 and its surrounding sequences, possesses the phosphatase holoenzyme inhibitory domain termed PHIN [6]. Other CPI-17-related proteins, including PP1 holoenzyme inhibitor (PHI-1) [7], kinase C-enhanced PP1 inhibitor (KEPI) [8], and gastrointestinal- and brain-specific PP1 inhibitor (GBPI-1) [9], have been identified, and these proteins include a PHIN domain with partial sequence homology to CPI-17 [4]. However, CPI-17 does not include the canonical RVxF PP1c binding motif, whereas this motif is present in PHI-1, KEPI, and GBPI-1. However, within the PHIN domain, the short sequence, including the Thr inhibitory phosphorylation site and surrounding residues, shows high homology between CPI-17 and the related proteins (PHI-1, KEPI, and GBPI-1) (see Table 1). Mutation analysis established that in addition to the phosphorylated Thr38 in CPI-17, Tyr41, Asp42, and Arg43/Arg44 are also necessary for the inhibitory activity [6], and the ^41^Tyr-Asp-Arg^43^ sequence motif is present in all family members. Tyr41 prevents the dephosphorylation of phospho-CPI-17 (P-CPI-17) by MP, and Arg43/Arg44 ensures the ionic interaction of CPI-17 with Asp5 of MYPT1, contributing to the specificity of CPI-17 toward the MP holoenzyme [10].

The role of CPI-17 in regulating the phosphorylation level of the 20 kDa myosin light chain and the contraction/relaxation cycle of smooth muscles has been characterized in detail [11]. Other non-muscle-related cellular effects of CPI-17 and related proteins have also been established. Merlin, a tumor suppressor, is dephosphorylated and activated by MP; however, the inhibition of MP by CPI-17 induces tumorigenic transformation in non-tumorigenic NIH 3T3 cells [12]. CPI-17 drives oncogenic Ras signaling in human melanomas [13] and is upregulated in schwannoma [14,15]. KEPI may be involved in the host response to SARS-CoV and in driving pathogenesis through TNFα signaling in SARS-CoV infections in mice [16]. In breast cancer cell lines and tissues, the downregulation of KEPI is implicated in the regulation of the tumor suppressor EGR1 via the MEK-ERK pathway [17]. PHI-1 is a pan-cancer marker regulating Raf-1 proteostasis [18]. The predicted role of PHI-1 in multiple cancers has been reviewed recently [19]. Based on these studies, CPI-17 and its related proteins play important roles in the regulation of numerous cellular processes in normal and diseased cells and tissues.

The phosphorylation-dependent inhibitory action of CPI-17 is reversible via dephosphorylation with protein phosphatases; however, controversial results have been presented on the identity of the enzymes responsible for dephosphorylation. Initial in vitro studies [20] suggested that type 2A (PP2A), -2B (PP2B), and -2C (PP2C) phosphatases dephosphorylate phospho-Thr38 (P-Thr38) in CPI-17, but not PP1. In contrast, a report showed [21] that CPI-17 was dephosphorylated by the glycogen-bound PP1 holoenzyme, but not by MP. In permeabilized rabbit femoral arteries, calyculin A (CLA, 1 µM), a phosphatase inhibitory toxin specific to both PP1 and PP2A, induced phosphorylation of CPI-17, but PP2A-specific inhibitors (okadaic acid and fostriecin) were ineffective. Therefore, the conclusion was drawn that, in arteries, PP1 holoenzymes distinct from MP may be the major CPI-17 phosphatases.

In the present study, we examined several cancerous cell lines for phosphorylated CPI-17-like proteins. After the induction of phosphorylation via CLA treatment, followed by Western blotting of cell lysates with anti-CPI-17^pThr38^, we did not identify phosphorylated CPI-17 or its related proteins (KEPI, PHI-1, or GBPI-1), but found phosphorylated protein signals predominantly in the 60–80 kDa range. Among these proteins, LIMK2-1 was identified. Our results imply that LIMK2-1 is a phosphorylation-dependent inhibitor of PP1.

## 2. Results

### 2.1. CLA Induces Phosphorylation of CPI-17-like Sequences of Proteins in HeLa and MCF7 Cells

**Figure 1:**
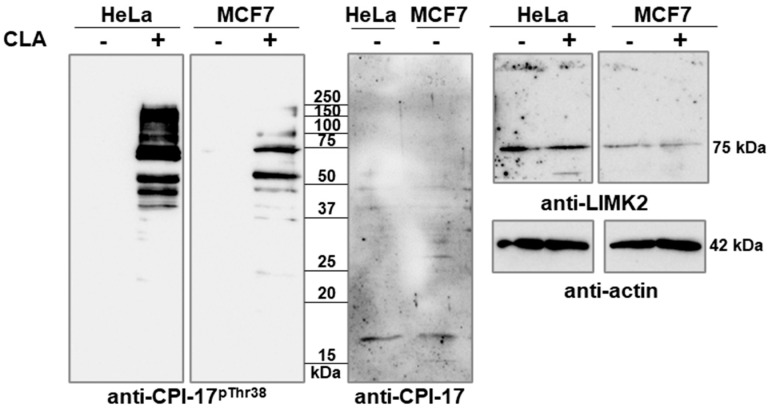
CLA induces phosphorylation of CPI-17-related proteins. Whole lysates of control and CLA-treated HeLa and MCF7 cells were analyzed via Western blotting using anti-CPI-17^pThr38^, anti-CPI-17, anti-LIMK2, and anti-actin antibodies.

In previous studies [22], we demonstrated that the treatment of THP-1 cells with CLA (50 nM) induced the phosphorylation of ERK1/2 and Akt kinases and a KEPI-like protein. The latter was identified with an anti-phospho-Thr38-CPI-17 antibody (anti-CPI-17^pThr38^). Activity assays specific to PP1 and PP2A established that PP2A activity was predominantly suppressed under these conditions (i.e., the treatment of cells with 50 nM CLA). These data imply that PP2A may be a phosphatase for P-CPI-17 and its related proteins under cellular conditions. We assayed lysates of CLA-treated HeLa and MCF7 cells for cross-reaction with anti-CPI-17^pThr38^ on Western blots (Figure 1, left panels). In the absence of CLA, we did not detect CPI-17-like phosphorylation sequences. Strikingly, in the presence of CLA, cross-reactions were obtained in the ~45–220 kDa range in HeLa cells and in the 60–80 kDa range in MCF7 cells, but not at the molecular mass levels of CPI-17 or its related proteins, although CPI-17 was present in both cell lines (Figure 1, middle panel). As a control for CLA-induced phosphorylation of other signaling proteins, a significant increase in the phosphorylation of p38 kinase was observed (Figure S1).

These results suggest that the proteins phosphorylated at a CPI-17-like phosphorylation site might fulfill the PP1 inhibitory function in the absence of phosphorylation of CPI-17 and its related proteins. To identify the proteins that cross-reacted with the antibody by recognizing the phospho-specific form of CPI-17, we performed a sequence similarity search. The results of the database search using the phosphorylation sequence of CPI-17 (^35^ARVTVKYDRREL^46^) are shown in Table 1. The highest identities (58–75%) with the CPI-17 phosphorylation sequence were found in PHI-1, KEPI, and GBPI-1, followed by LIMK2 (58%) and PSKH1 (58%). Other proteins have 50% or less identity. Among the possible CPI-17-like proteins, LIM kinase 2 isoform 1 (LIMK2-1) was identified at the ~75–78 kDa molecular mass, with a LIMK2-specific antibody in HeLa and MCF7 cells (Figure 1, right panels). No proteins in the higher molecular mass range (>80 kDa) phosphorylated in HeLa cells were detected via anti-CPI-17^pThr38^. The reasons for this discrepancy are not known.

### 2.2. Phosphorylation of Flag-LIMK2-1 at the CPI-17-like Sequences

**Figure 2:**
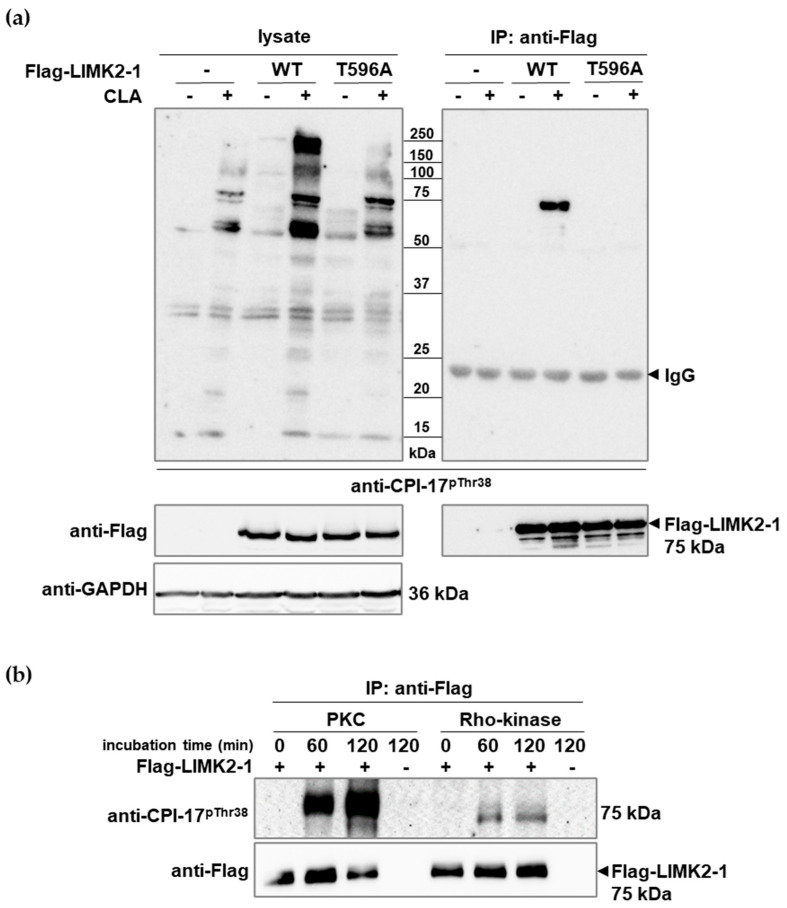
Phosphorylation of Flag-LIMK2-1 at the CPI-17-like sequence. (**a**) TsA201 cells expressing wild-type Flag-LIMK2-1 (WT) or Flag-LIMK2-1^T596A^ (T596A) were treated with CLA, and immunoprecipitation was performed using anti-Flag affinity resin. Cell lysates and resin-bound proteins were analyzed via Western blotting using anti-CPI-17^pThr38^, anti-Flag, or anti-GAPDH antibodies. (**b**) Flag-LIMK2-1 immobilized on the anti-Flag affinity resin was phosphorylated by PKC or Rho-kinase, as described in Materials and Methods. Phosphorylation of Flag-LIMK2-1 was detected via Western blotting using anti-CPI-17^pThr38^ antibody. Protein loading was verified using an anti-Flag antibody.

Flag-LIMK2-1 or its phosphorylation site mutant (T596A) was expressed in tsA201 cells, and the cells were untreated or treated with CLA. Cell lysates were assayed with anti-CPI-17^pThr38^ on Western blotS (Figure 2a, left panel). Without Flag-LIMK2-1 overexpression, tsA201 cells exhibited similar protein phosphorylation patterns to HeLa and MCF7 cells (compare to Figure 1) after CLA treatment, and this pattern included the 75 kDa protein suspected to be LIMK2-1 (see Figure S2). Overexpression of wild-type or mutant Flag-LIMK2-1 (Flag-LIMK2-1^WT^ or Flag-LIMK2-1^T596A^) in tsA201 cells enhanced phosphorylation levels at 75 kDa, which was more apparent for the wild type. No significant phosphorylation signals were detected in the absence of CLA. Immunoprecipitation with anti-Flag coupled resins was performed, and the eluted proteins were analyzed on Western blots exposed to anti-CPI-17^pThr38^ or anti-Flag antibodies (Figure 2a, right panel). Cross-reaction with anti-CPI-17^pThr38^ occurred only with Flag-LIMK2-1^WT^ in the presence of CLA, but not with Flag-LIMK2-1^T596A^, indicating the necessity of phosphorylation on Thr596 of LIMK2-1 to be recognized by the phospho-CPI-17-specific antibody.

Among the CPI-17 kinases, protein kinase C (PKC) and RhoA-activated kinase (Rho-kinase) have major physiological significance [4,11]. Therefore, we applied these two kinases in purified forms to probe the phosphorylation of anti-Flag precipitated Flag-LIMK2-1 in vitro. Figure 2b shows that PKC phosphorylated Flag-LIMK2-1 at the CPI-17-like phosphorylation sequence, but Rho-kinase appeared to be less effective.

**Figure 3:**
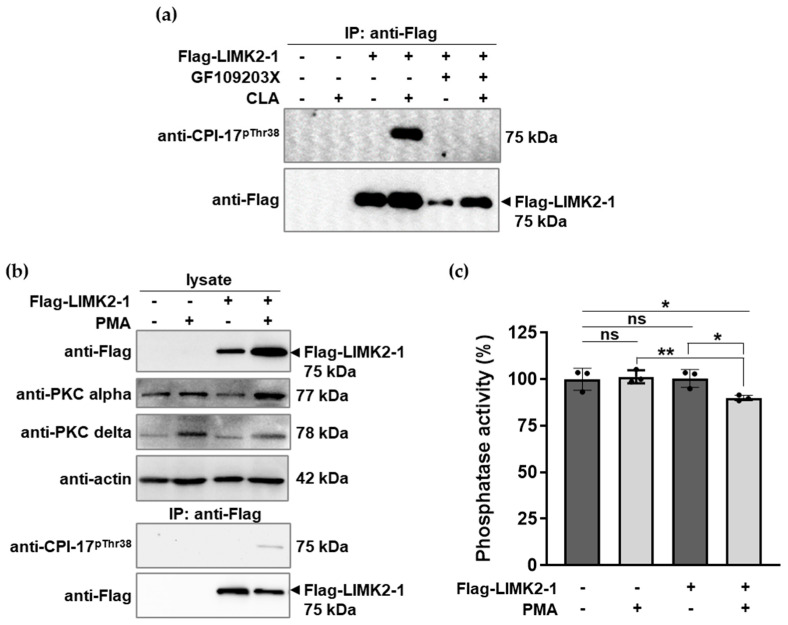
Phosphorylation of LIMK2-1 induced via phosphatase inhibition and kinase activation in tsA201 cells expressing Flag-LIMK2-1. (**a**) TsA201 cells expressing Flag-LIMK2-1 were treated with GF109203X, followed by incubation in the absence or presence of CLA. Immunoprecipitates were analyzed via Western blotting using anti-CPI-17^pThr38^ or anti-Flag antibodies. (**b**,**c**) TsA201 cells were transfected with empty or Flag-LIMK2-1-expressing vectors and treated with PMA. (**b**) Cell lysates and immunoprecipitates were analyzed via Western blotting using anti-Flag, anti-PKC alpha, anti-PKC delta, anti-actin, or anti-CPI-17^pThr38^ antibodies. (**c**) Phosphatase activity of cell lysates was determined using a ^32^P-MLC20 substrate and expressed as a percentage of the untreated empty vector-expressing control. Values are means ± SD. Statistical analysis was performed using multiple *t*-tests (*n* = 3, ns, not significant; * *p* < 0.05; ** *p* < 0.01).

To establish the role of PKC in the phosphorylation of Flag-LIMK2-1 overexpressed in tsA201 cells, we tested the effects of GF109203X, a potent PKC inhibitor compound, on CLA-induced phosphorylation. Figure 3a shows that the PKC inhibitor completely inhibited the phosphorylation of Flag-LIMK2-1. The role of PKC was also assessed by treating the cells with phorbol 12-myristate 13-acetate (PMA) to induce the PKC-dependent phosphorylation of possible target proteins. Figure 3b illustrates that PKCα and PKCδ were present in tsA201 cells. In the empty vector transfected cells, no LIMK2-1 phosphorylation was detected either in the presence or absence of PMA. In contrast, in Flag-LIMK2-1-expressing tsA201 cells, PMA induced the phosphorylation of the CPI-17-like sequences, as revealed via Western blotting of the anti-Flag precipitates with the anti-CPI-17^pThr38^ antibody (Figure 3b, lower panel). These results confirm that LIMK2-1 phosphorylation at the CPI-17-like site is PKC-dependent and induced either via phosphatase inhibition using CLA (Figure 3a) or via the activation of PKC using PMA under cellular conditions in Flag-LIMK2-1-expressing cells (Figure 3b). The influence of Flag-LIMK2-1 phosphorylation in tsA201 cells on the activity of cellular phosphatases was also of interest. Therefore, phosphatase activity in the lysates of the untreated and PMA-treated cells was assayed using a ^32^P-MLC20 substrate (Figure 3c). PMA treatment did not influence the phosphatase activity in tsA201 cells transfected with the empty vector. In contrast, in Flag-LIMK2-1-transfected cells, PMA treatment caused a slight but significant decrease in phosphatase activity compared to the untreated cells. These results (Figure 3b,c) imply that the phosphorylation of CPI-17-like sequences in LIMK2-1 is coupled with the inhibition of protein phosphatase activity.

### 2.3. LIMK2-1 Interacts with and Inhibits PP1c and Reconstituted Myosin Phosphatase

**Figure 4:**
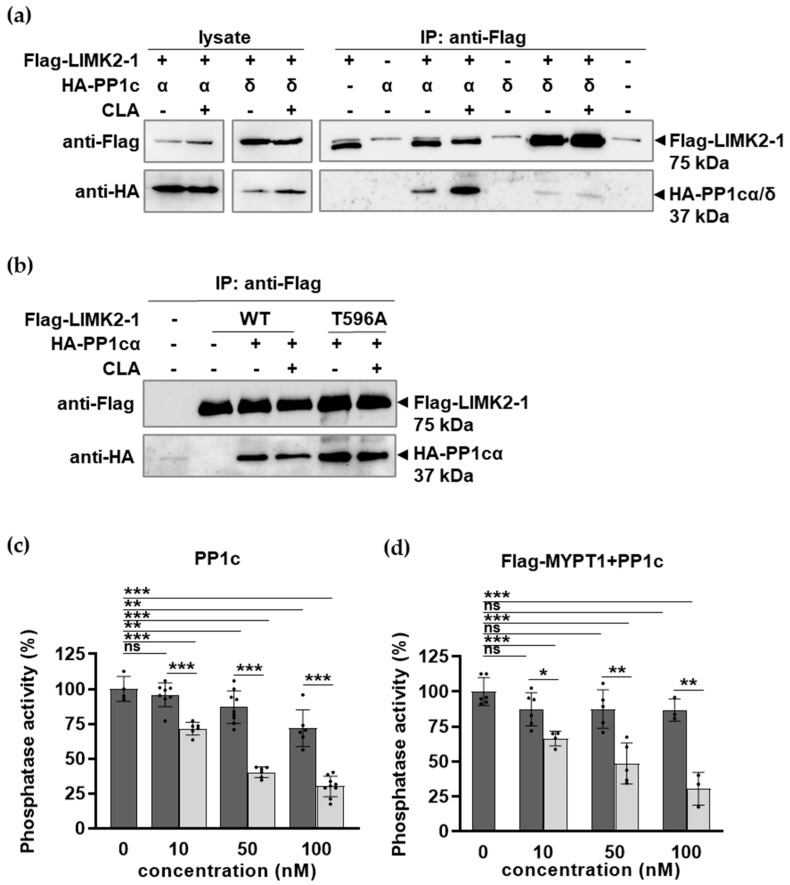
Phosphorylated LIMK2-1 interacts with and inhibits PP1. (**a**,**b**) Immunoprecipitation with anti-Flag affinity resin from tsA201 cell lysates expressing Flag-LIMK2-1 (wild type (WT) in (**a**,**b**)) or Thr596 mutant (T596A, only in (**b**)), HA-PP1cα/δ or Flag-LIMK2-1 + HA-PP1cα/δ after CLA treatment. Cell lysates and resin-bound proteins were analyzed via Western blotting using anti-Flag or anti-HA antibodies. (**c**,**d**) Flag-LIMK2-1 (dark columns) or thiophosphorylated Flag-LIMK2-1 (light columns) was incubated with native PP1c (**c**) or PP1c + Flag-MYPT1 holoenzyme (**d**), and phosphatase activity was determined using ^32^P-MLC20 substrate, as described in Materials and Methods. Phosphatase activity of samples without Flag-LIMK2-1 was set at 100%. Statistical analysis was performed using multiple *t*-test (*n* = 4–8, ns, not significant; * *p* < 0.05; ** *p* < 0.01; and *** *p* < 0.001).

We investigated the possible interactions between non-phosphorylated and phosphorylated Flag-LIMK2-1 (induced by CLA treatment) and PP1c in tsA201 cells. HA-tagged PP1cα and -PP1cδ were co-expressed with Flag-LIMK2-1, immunoprecipitated on anti-Flag antibody resin, and the bound proteins were analyzed with anti-HA and anti-Flag antibodies. (Figure 4a). For an unknown reason, the expression of HA-PP1cδ was relatively low compared to that of HA-PP1cα. Nevertheless, both HA-PP1cα and HA-PP1cδ co-precipitated with both non-phosphorylated and phosphorylated Flag-LIMK2-1. We did not detect MYPT1 in the precipitates using an anti-MYPT1 antibody.

The interactions of Flag-LIMK2-1^WT^ and Flag-LIMK2-1^T596A^ with PP1cα with co-expression and co-precipitation were compared as described in Figure 4a. Figure 4b demonstrates that HA-PP1cα co-precipitated with both Flag-LIMK2-1^WT^ and Flag-LIMK2-1^T596A^, implying that phosphorylation is not required for the interaction of LIMK2-1 with PP1c, and that mutation of the phosphorylation site (T596A) does not interfere with the interaction.

Flag-LIMK2-1 was expressed in tsA201 cells and isolated on anti-Flag resin, then eluted by Flag peptide to prepare non-phosphorylated Flag-LIMK2-1. Alternatively, the anti-Flag resin-isolated Flag-LIMK2-1 was thiophosphorylated by PKC and then eluted with the Flag peptide. These non-phosphorylated and thiophosphorylated Flag-LIMK2-1 were used in phosphatase assays to determine their influence on the activity of native PP1c (Figure 4c) or MP reconstituted from native PP1c plus Flag-MYPT1 (Figure 4d). Figure 4c illustrates that a slight but significant inhibition of PP1c occurred using the non-phosphorylated Flag-LIMK2-1, but thiophosphorylated Flag-LIMK2-1 induced a more pronounced decrease in the phosphatase activity, with an IC50 of ~28 nM. Similar influences were observed for the effects of Flag-LIMK2-1 on the activity of the MP holoenzyme (Figure 4d), including non-significant inhibition by the non-phosphorylated Flag-LIMK2-1 and less inhibitory effects (IC50 of ~47 nM) from the thiophosphorylated Flag-LIMK2-1 compared to the effects of PP1c (Figure 4c). These results suggest that LIMK2-1 inhibits the activity of PP1c and MP and its inhibitory effectiveness increases via the phosphorylation of the CPI-17-like sequences.

## 3. Discussion

Our results include two novel findings in the protein phosphatase field. In malignant cells (HeLa and MCF7), the phosphorylation of proteins in the range of 40–220 kDa was detected in CPI-17-like phosphorylation sequences after the induction of phosphorylation by the phosphatase inhibitor CLA and detection with anti-CPI-17^pThr38^. This result suggests that these proteins might function as PP1 inhibitors, and LIMK2 was identified as a phosphorylation-dependent inhibitor of PP1c and MP holoenzyme. The sequence similarity between CPI-17 and LIMK2 was previously reported [23], but the effects of LIMK2 on phosphatase activity were not investigated. Recent work [24] indicated that among the LIMK2 isoforms (LIMK2a, LIMK2b, and LIMK2-1), only LIMK2-1 includes the phosphorylation sequence (LIMK2-1^593–604^) similar to CPI-17 and PHI-1. A KVRF PP1c binding motif is present in all LIMK2 isoforms. In addition, the interaction of LIMK2-1 with PP1c was proven and thought to be involved in the inhibition of cofilin dephosphorylation. However, the phosphorylation of LIMK2-1 at Thr596 was not shown. Our present data imply that non-phosphorylated LIMK2-1 slightly inhibits PP1c, but that the phosphorylation of LIMK2-1 at Thr596 profoundly increases the inhibitory effectiveness towards PP1c and MP (IC50 ~28 and ~47 nM, respectively). These results suggest that, as a phosphatase inhibitor, LIMK2-1 is similar to the CPI-17-related proteins (PHI-1, KEPI, and GBPI-1). LIMK2-1 includes a consensus RVxF PP1c binding motif in addition to the CPI-17-like phosphorylation sequence.

The 12-amino-acid sequence, including the inhibitory Thr596 phosphorylation site of LIMK2-1, exhibits 58% identity with CPI-17. In addition to the Thr596 phosphosite of LIMK2-1, Tyr599 is also present, and this residue is important in preventing the dephosphorylation of phospho-Thr596 via MP [6]. Flag-LIMK2-1 interacts with PP1cα and PP1cδ in both the non-phosphorylated and phosphorylated states, as shown by the anti-Flag co-immunoprecipitation assays. The mutation of Thr596 to Ala did not influence this interaction. Although phospho-LIMK2-1 inhibits MP with similar potency to PP1c, we could not detect any interaction with MYPT1. In CPI-17, Arg43/44 may interact with Asp5 in MYPT1 in the N-terminal region [10]. In LIMK2-1, the C-terminal to the P-Thr596 inhibitory site, these residues are Pro601 and Lys602; their interaction (Pro601/Lys602) with the N-terminal MYPT1 region may be, if at all, weaker than the CPI-17 counterpart (Arg43/44), and other steric interference cannot be excluded.

The phosphorylation status of cellular proteins is balanced by the activity of protein kinase(s) and phosphatase(s) acting on the phosphorylated targets. Protein phosphorylation may be increased by the activation of the protein kinases and/or inhibition of the protein phosphatases. Of note, the phosphorylation of LIMK2-1 and other unidentified proteins is enhanced substantially by phosphatase inhibition with CLA. The phosphatase type, inhibited under our applied conditions, is presumably PP2A, as it was previously reported that 50 nM of CLA predominantly suppresses this enzyme [22]. Our in vitro assays suggest that PKC and Rho-kinase are involved in the phosphorylation of the Thr596 residue in LIMK2-1. In addition, the role of PKC in this phosphorylation under cellular conditions was demonstrated in PMA-treated cells overexpressing LIMK2-1. The PMA-induced phosphorylation of LIMK2-1 was accompanied by reduced phosphatase activity, implying a phosphatase inhibitory function of phosphorylated LIMK2-1. Of note, the extent of the PMA-induced phosphorylation of Flag-LIMK2-1 expressed in tsA201 cells is relatively low and results in modestly decreased phosphatase activity. In contrast, CLA induces the robust phosphorylation of native LIMK2-1 and other CPI-17-like proteins in HeLa and MCF7 cells. These results indicate that other kinases, yet unidentified, may also phosphorylate proteins, including the CPI-17-like PP1c and MP inhibitory phosphorylation sequences.

In conclusion, our results indicate that in certain cancer cell lines where phosphorylated CPI-17 or its related proteins (PHI-1, KEPI, or GBPI-1) are not detected, proteins with a molecular mass of 40–220 kDa range are phosphorylated in CPI-17-like sequences that are cross-reactive with anti-CPI-17^pThr38^. Among these proteins, we identified LIMK2-1 as a phosphorylation-dependent inhibitor of PP1c and MP. MP plays a role in the dephosphorylation and activation of tumor suppressor proteins, which is counterbalanced by inhibition with phospho-CPI-17 [12,13]. MP is ubiquitously expressed in mammalian cells and has widespread cellular functions [25]. LIMK2-1 or other phosphorylated proteins yet to be identified regulate MP in cells and may be the subject of more detailed studies.

## 4. Materials and Methods

### 4.1. Proteins and Antibodies

^32^P-labeled myosin light chain (^32^P-MLC20), the catalytic subunit of protein phosphatase-1 (PP1c) and the Flag-tagged form of myosin phosphatase target subunit 1 (Flag-MYPT1), were prepared as previously described [26,27]. Anti-CPI-17^pThr38^ was a generous gift from Matsumi Eto (Okayama University of Science, Imabari, Ehime, Japan). The sources of other antibodies were as follows: anti-Flag (#20543-1-AP, Proteintech, Rosemont, IL, USA); anti-HA (#71-5500, Invitrogen, Waltham, MA, USA); anti-CPI-17 (#07-344, Merck, Darmstadt, Germany); anti-LIMK2-1 (#H00003985-D01P, Abnova, Taipei, Taiwan); anti-p38 MAPK^pThr180/Tyr182^ (#9211, Cell Signaling Technology, Danvers, MA, USA); anti-PKC Alpha (#21991-1-AP, Proteintech); anti-PKC delta (#2058, Cell Signaling Technology); anti-actin-HRP (#sc-47778, Santa Cruz Biotechnology, Dallas, Texas USA); anti-GAPDH (#sc-47724, Santa Cruz Biotechnology); Clean-Blot IP Detection Reagent (#21230, Thermo Fisher Scientific, Waltham, MA USA); anti-rabbit IgG (#A0545, Sigma-Aldrich, St. Louis, MO, USA); anti-mouse IgG (#A2554, Sigma-Aldrich); and anti-chicken IgY (#A9046, Sigma-Aldrich).

### 4.2. Cell Cultures and Treatments

HeLa (human cervical carcinoma cells), MCF7 (human Caucasian breast adenocarcinoma), and tsA201 (transformed human kidney, identical to 293 cell line) cells were purchased from the European Collection of Cell Cultures (ECACC, Salisbury, UK) and cultured in DMEM supplemented with 2 mM of L-glutamine, 100 U/mL of penicillin/streptomycin, 1% non-essential amino acids (only for MCF7), and 10% FBS. Cells were treated with 50 nM of calyculin A (CLA, Calbiochem, San Diego, CA, USA) for 30 min or 5 µM of GF109203X (GF, Tocris, Bristol, UK) for 30 min, followed by incubation in the absence or presence of CLA for another 30 min. Treatments with phorbol 12-myristate 13-acetate (PMA, Tocris) were performed for 30 min at a final concentration of 1 µM. Control cells were treated with 0.1% DMSO (vehicle control).

For Western blotting, cell proteins were precipitated using the trichloroacetic acid/acetone protocol and boiled in SDS sample buffer. Alternatively, cell extracts were prepared in lysis buffer (50 mM of Tris-HCl pH 7.4 with 150 mM of NaCl, 1 mM of EDTA, and 1% Triton X-100, freshly supplemented with 0.5% protease inhibitor cocktail (#S8830, Sigma-Aldrich), 1 mM of NaF, 1 mM of Na_4_P_2_O_7_, 1 mM of β-glycerophosphate, 1 mM of Na_3_VO_4_, and 1 μM of microcystin-LR (gift from Csaba Mathe, Department of Botany, University of Debrecen, Hungary), and applied for immunoprecipitation and subsequent Western blotting. For the phosphatase assay, cells were lysed in Tris-buffered saline (TBS) pH 7.4, containing 0.1 mM of EDTA and 0.5% protease inhibitor cocktail.

### 4.3. Expression Plasmids and Site-Directed Mutagenesis

The pReceiver-M11/Flag-LIMK2-1 vector encoding the human full-length LIMK2 isoform 1 (LIMK2-1; NM_001031801.1) with an N-terminal Flag tag was purchased from GeneCopoeia (EX-Z0703-M11, GeneCopoeia, Rockville, MD, USA). The Thr596 mutant of LIMK2-1 was created via site-directed mutagenesis using Quick-Change II XL Site-Directed Mutagenesis Kit (Agilent Technologies, Santa Clara, CA, USA) with the following primers: 5′-G GTC ATA CTT GAT **GGC** GAC CTT CCC TTG GCG-3′ (forward) and 5′-CGC CAA GGG AAG GTC **GCC** ATC AAG TAT GAC C-3′ (reverse). The mutation was verified via DNA sequencing (UD-GenoMed Medical Genomic Technologies Ltd., Hungary) and is referred to as LIMK2-1^T596A^. The expression of PP1cα and PP1cδ was achieved using pcDNA3.1(+)-N-HA/PP1cα and pcDNA3.1(+)-N-HA/PP1cδ (Genscript, Piscataway, NJ, USA) plasmids, respectively. Control cells were transfected with pcDNA3.1+N-DYK empty vector (GenScript).

### 4.4. Cell Transfections

TsA201 cells were subcultured the day before transfection, and the plasmid was introduced into cells using polyethyleneimine transfection reagent (Polyplus, Illkirch, France) according to the manufacturer’s instructions. After 48 h, cells were treated and lysed, and the overexpression was verified by Western blotting using anti-Flag and anti-HA antibodies.

### 4.5. Immunoprecipitation and Purification of Flag-LIMK2-1

For the immunoprecipitation of Flag-LIMK2-1, tsA201 cell lysate was added to anti-Flag M2 affinity resin (#A2220, Sigma-Aldrich) and incubated for 2 h at 4 °C with continuous rotation. After centrifugation, the unbound lysate was removed, the resin was washed three times with TBS, and the resin-bound proteins were eluted with SDS sample buffer or TBS containing 300 μg/mL of Flag peptide (Sigma-Aldrich), 2 M of MgCl_2_, and 0.5% protease inhibitor mix. Protein samples were desalted using a Zebra Spin desalting column (Thermo Fisher Scientific).

### 4.6. Western Blotting

Cell lysates and immunoprecipitated (IP) samples were loaded onto 10 or 12% SDS-polyacrylamide gels and analyzed via immunoblotting, as described earlier [22]. For the detection of target proteins in IP samples, Clean-blot IP detection reagent was used, which can detect primary antibodies without interference from denatured IP antibodies.

### 4.7. In Vitro Phosphorylation

Flag-LIMK2-1 immobilized on anti-Flag M2 affinity resin was phosphorylated using 0.1 μg/mL of PKC (#V526A, Promega, Madison, WI, USA) in Hepes buffer (20 mM of Hepes (pH 7.5), 10 mM of MgCl_2_, and 1 mM of DTT) in the presence of phosphatidylserine-diolene micelles (0.3 mg/mL of phosphatidylserine and 60 μg/mL of diolein), 0.65 mM of CaCl_2_, 1 μM of MC-LR, and 0.2 mM of ATP or 0.2 mM of ATPγS. For phosphorylation via Rho-kinase (#14-338, Merck), 2 U/mL of enzyme was applied in 50 mM of Tris-HCl (pH 7.5), with 0.1 mM of EGTA, 5 mM of MgCl_2_, 1 μM of MC-LR, and 0.2 mM of ATP. Samples without enzyme or Flag-LIMK2-1 substrates were used as negative controls. Reactions were carried out at 30 °C for 0, 60, or 120 min; the resins were washed, the phosphorylated proteins were eluted, and the buffer was exchanged, as described above. The purity of protein samples was checked using Coomassie Brilliant Blue staining after SDS-PAGE.

### 4.8. Protein Phosphatase Assay

To determine the phosphatase inhibitory effect, ^32^P-labeled MLC20 (^32^P-MLC20) was used as a substrate [26]. The effect of Flag-LIMK2-1 on PP1 activity was determined using native PP1c. PP1c (5 nM) diluted in 20 mM of Tris-HCl buffer (pH 7.4) containing 0.1% BSA and 0.1% 2-mercaptoethanol was incubated with 0-100 nM of Flag-LIMK2-1 or thiophosphorylated Flag-LIMK2-1 for 10 min at 30 °C in a volume of 20 μL. The reaction was started by the addition of 10 µL of substrate, incubated for 10 min, and stopped by the addition of 200 µL of 10% TCA and 200 µL of 6 mg/mL BSA. Precipitated proteins were collected via centrifugation, and the released ^32^Pi was determined from the supernatant in a scintillation counter (Perkin Elmer, Waltham, MA, USA). For holoenzyme measurements, 5 nM of PP1c was mixed with 5 nM of purified Flag-MYPT1 and pre-incubated for 10 min before the addition of Flag-LIMK2-1. Phosphatase activity of samples not containing Flag-LIMK2-1 was set at 100%. Phosphatase activities of minimally diluted tsA201 cell lysates were assayed with ^32^P-MLC20 for 1 min and expressed as a percentage of control (empty vector-transfected, untreated sample). Statistical analyses were performed using multiple *t*-tests with GraphPad Prism9 software.

### 4.9. Sequence Similarity Search

To identify proteins containing the CPI-17 phosphorylation sequence, we applied the Basic Local Alignment Search Tool (BLAST) developed by NCBI. The amino acid sequence ARVTVKYDRREL was specified as the query and default settings, and algorithm parameters were used. The search was restricted to human sequences. The search results were validated using the Clustal Omega multiple sequence alignment program (https://www.ebi.ac.uk/jdispatcher/msa/clustalo?stype=protein (accessed on 27 July 2025); EMBL-EBI). Sequences with significant similarities were aligned with the query sequence, conserved amino acids were identified, and the degree of similarity was calculated as a percentage of identical amino acids. Information on the length and molar mass of the identified proteins was obtained from the Uniprot protein database.

## Figures and Tables

**Table 1:** CPI-17 phosphorylation site similarities in human proteins: identities are highlighted in blue.

	Gene Name	Protein Name	Accession Number	Amino Acids	MW (Da)	Identities	Sequence											
1.	PPP1R14A	Protein phosphatase 1 regulatory subunit 14A (CPI-17)	NP_150281.1 NP_001230876.1	147 120	16,693 13,480	12/12	A	R	V	T	V	K	Y	D	R	R	E	L
2.	PPP1R14B	Protein phosphatase 1 regulatory subunit 14B (PHI-1)	NP_619634.1	147	15,911	9/12 (75%)	G	K	V	T	V	K	Y	D	R	K	E	L
3.	PPP1R14C	Protein phosphatase 1 regulatory subunit 14C (KEPI)	NP_112211.1	165	17,843	9/12 (75%)	G	K	V	T	V	K	Y	D	R	K	E	L
4.	PPP1R14D	Protein phosphatase 1 regulatory subunit 14D isoform (GBPI-1)	NP_060196.1 NP_001123615.1	145 200	16,508 22,430	7/12 (58%)	S	R	L	T	V	K	Y	D	R	G	Q	L
5.	LIMK2	LIM kinase 2	NP_001026971.1 AAB54055.1 KAI2597385.1	686 733 629	77,886 no data 71,193	7/12 (58%)	G	K	V	T	I	K	Y	D	P	K	E	L
6.	PSKH1	Serine/threonine-protein kinase H1	NP_006733.1	424	48,035	7/12 (58%)	P	R	V	T	A	K	Y	D	I	K	A	L
7.	TIGAR	Fructose-2,6-bisphosphatase TIGAR	NP_065108	270	30,063	6/12 (50%)	K	D	M	T	V	K	Y	D	S	R	L	R
8.	BACE2	Beta-secretase 2 (memapsin 1)	NP_036237.2	518	56,180	5/12 (42%)	F	D	V	T	V	K	Y	T	Q	G	S	W
9.	LIPK	Lipase family member K	NP_001073987.1	399	45,563	5/12 (42%)	P	V	V	T	V	K	Y	T	Q	S	P	M
10.	PM20D2	Xaa-Arg dipeptidase	NP_001010853.1	436	47,776	5/12 (42%)	H	D	V	T	V	K	Y	Y	G	K	A	S
11.	FAM210A	Protein FAM210A	NP_001092271.1	272	30,777	6/12 (50%)	T	S	V	T	V	K	Y	L	R	S	H	G

## Data Availability

The original contributions presented in this study are included in the article/Supplementary Material. Further inquiries can be directed to the corresponding authors.

## Supplementary Materials

The following supporting information can be downloaded at: https://www.mdpi.com/article/10.3390/ijms26157347/s1, Original Western blots images.

## Abbreviations

The following abbreviations are used in this manuscript:

CLA	Calyculin A
CPI-17	Protein kinase C-activated protein phosphatase-1 inhibitor of 17 kDa
Flag-LIMK2-1	Flag-tagged LIM kinase 2 isoform 1
GBPI-1	Gastrointestinal- and brain-specific PP1 inhibitor
HA-PP1cα	HA-tagged protein phosphatase 1 catalytic subunit α isoform
HA-PP1cδ	HA-tagged protein phosphatase 1 catalytic subunit δ isoform
KEPI	Kinase C-enhanced PP1 inhibitor
LIMK2	LIM kinase 2
LIMK2-1	LIM kinase 2 isoform 1
MLC20	20 kDa light chain of myosin
^32^P-MLC20	^32^P-labeled MLC20
MP	Myosin phosphatase
MYPT1	Myosin phosphatase target subunit-1
PHI-1	Pphosphatase holoenzyme inhibitor-1
PKC	Protein kinase C
PMA	Phorbol 12-myristate 13-acetate
PP1	Protein phosphatase-1
PP1c	PP1 catalytic subunit
PP1cα	PP1 catalytic subunit α isoform
PP1cδ	PP1 catalytic subunit δ isoform
