# Supplementary material for: LIMK2-1 Is a Phosphorylation-Dependent Inhibitor of Protein Phosphatase-1 Catalytic Subunit and Myosin Phosphatase Holoenzyme
Source: Int J Mol Sci. 2025 Jul 30;26(15):7347. doi: 10.3390/ijms26157347 (PMC12347249; doi:10.3390/ijms26157347)
Supplement: Supplementary file 1 [file ijms-26-07347-s001.zip › ijms-3767940-supplementary.pdf]

Figure S1

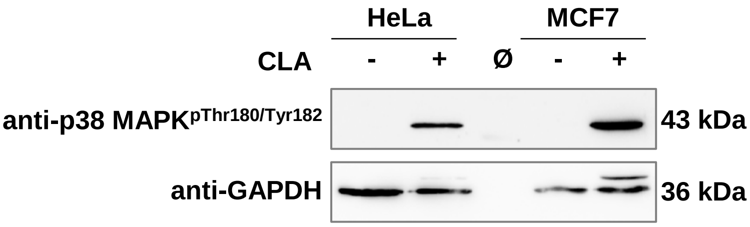

Figure S1. CLA induces phosphorylation of p38 MAPK in HeLa and MCF7 cells. Whole cell lysates of control and CLA-treated HeLa and MCF7 cells were analyzed by Western blotting using anti-p38 MAPK$^{pThr180/Tyr182}$ and anti-GAPDH antibodies.

Figure S2

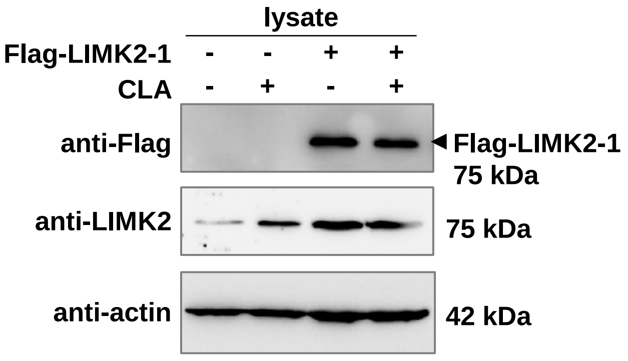

Figure S2. Analysis of LIMK2 in cell lysates used for IP experiments. Untransfected or Flag-LIMK2-1-transfected tsA201 were treated with none or CLA. Cell lysates were analyzed by Western blot using anti-Flag, anti-LIMK2 and anti-actin antibodies.
